# Good Practice for Conference Abstracts and Presentations: GPCAP

**DOI:** 10.1186/s41073-019-0070-x

**Published:** 2019-06-05

**Authors:** Cate Foster, Elizabeth Wager, Jackie Marchington, Mina Patel, Steve Banner, Nina C. Kennard, Antonia Panayi, Rianne Stacey, the GPCAP Working Group

**Affiliations:** 1 Watermeadow Medical, Ashfield Healthcare Communications, Witney, UK; 2 Sideview, Princes Risborough, UK; 3 University of Split, Split, Croatia; 4 Caudex, a McCann Health Company, Oxford, UK; 5 Alexion Pharmaceuticals Inc, New Haven, CT USA; 6 Darwin Grey Communications Ltd, Oxford, UK; 7 Cello Health MedErgy, a Cello Health PLC Company, Farnham, UK; 8 Shire International GmbH (now part of Takeda), Global Medical Affairs, Zug, Switzerland; 9 iMed Comms, Ashfield Healthcare Communications, Witney, UK

## Abstract

Research that has been sponsored by pharmaceutical, medical device and biotechnology companies is often presented at scientific and medical conferences. However, practices vary between organizations and it can be difficult to follow both individual conference requirements and good publication practice guidelines. Until now, no specific guidelines or recommendations have been available to describe best practice for conference presentations.

This document was developed by a working group of publication professionals and uploaded to PeerJ Preprints for consultation prior to publication; an additional 67 medical societies, medical conference sites and conference companies were also asked to comment. The resulting recommendations aim to complement current good publication practice and authorship guidelines, outline the general principles of best practice for conference presentations and provide recommendations around authorship, contributorship, financial transparency, prior publication and copyright, to conference organizers, authors and industry professionals.

While the authors of this document recognize that individual conference guidelines should be respected, they urge organizers to consider authorship criteria and data transparency when designing submission sites and setting parameters around word/character count and content for abstracts. It is also important to recognize that conference presentations have different limitations to full journal publications, for example, in the case of limited audiences that necessitate refocused abstracts, or where lead authors do not speak the local language, and these have been acknowledged accordingly. The authors also recognize the need for further clarity regarding copyright of previously published abstracts and have made recommendations to assist with best practice.

By following Good Practice for Conference Abstracts and Presentations: GPCAP recommendations, industry professionals, authors and conference organizers will improve consistency, transparency and integrity of publications submitted to conferences worldwide.

## Note on terminology

*Company* refers to any medical commercial organization involved with research, such as pharmaceutical or biotechnology companies and medical device manufacturers.

*Company-sponsored* refers to all types of research (preclinical and clinical, pre- and post-marketing) that is directly sponsored and/or funded by a company. While this classification does not necessarily include research performed under other types of funding arrangement, such as investigator-sponsored or investigator-initiated trials or research (where companies are not involved with conference presentations or publications), those involved in submitting investigator-initiated study material to conferences are encouraged to consider following these recommendations.

*Conference* is used to refer to meetings, often organized by academic societies, that invite submissions (usually as abstracts) presenting research findings on an aspect of medicine or science. Such conferences have a scientific (or programme) committee that reviews and selects presentations to be given at the meeting from the submitted abstracts.

*Abstract* refers to those submitted for consideration to scientific and medical conferences (see above).

*Presentation* refers to posters or slides developed from abstracts accepted for presentation at such conferences.

*Lead author* refers to the person who normally presents study findings at a conference and is usually listed as the first author. This is often the Principal Investigator.

*Society sponsor* refers to a member of the society that is holding the conference, who acts as sponsor (or guarantor) of a submitted abstract.

*Presenting author* refers to the person on the author list who attends the conference and presents the poster or abstract.

*Non-author presenter or local presenter* refers to a person who presents on behalf of the author group, but who is not listed as an author.

## Introduction

Research that has been sponsored (see the ‘Note on terminology’ section for precise definitions of these terms) by commercial organizations (e.g. pharmaceutical, medical device and biotechnology companies) is often presented at scientific and medical conferences. These conferences are pivotal for the presentation of data from ongoing research projects and clinical trials to the relevant audience and are often the first opportunity to disclose and discuss potentially practice-changing data. They facilitate early communication of data long before publication of a full manuscript and also provide the opportunity to present results of additional analyses such as secondary and/or exploratory endpoints and post hoc analyses. However, while abstracts submitted to conferences are reviewed by a scientific committee for suitability and interest to the audience prior to acceptance, it is important to note that they are not considered peer-reviewed as they are not subject to the same rigorous peer-review process as are journal articles. Poster and oral presentations based upon accepted abstracts are rarely, if ever reviewed. Furthermore, a recent systematic review showed that less than 50% of all studies accepted as abstracts went on to be published in full following presentation at a conference [1]. While it is desirable to strive for full publication after a conference presentation to ensure transparency and allow healthcare professionals to make appropriate informed decisions based on the peer-reviewed literature, this is not always practical and/or achievable. Therefore, it is important that abstracts and conference presentations, particularly for company-sponsored research, are developed with as rigorous a process as that of a full publication, because these may ultimately become the only source for a particular analysis.

While there are recommendations on the preparation of journal articles and qualification for authorship [2], and guidelines for best practices in the publication of company-sponsored research [3–5], until now, no specific guidelines have been available to describe good practice and best principles for conference presentations. This has resulted in diverse practices and a lack of standard expectations for transparency and ethical approaches. Although some aspects of good practice in Good Publication Practice (GPP) [5] and in reporting guidelines such as CONSORT and PRISMA for Abstracts [6, 7] can be applied to conference presentations, the most widely cited recommendations on authorship from the International Committee of Medical Journal Editors (ICMJE) relate exclusively to publications in peer-reviewed journals [3]. These recommendations were not designed for, and therefore are not fully applicable to, abstract submissions and conference presentations and are challenging to implement in practice. Building on the acceptance and recognition of the GPP guidelines (first published as GPP for Pharmaceutical Companies in 2003 [3], updated in 2010 [4] and most recently published as GPP3 in 2015 [5]), this article endeavours to extend their principles and to address challenges relating to the presentation of company-sponsored research at academic meetings. These recommendations, on Good Practice for Conference Abstracts and Presentations (GPCAP), focus on company-sponsored research (see the ‘Note on terminology’ section). However, they do not cover other company activities that may be linked to conferences (e.g. satellite symposia organized alongside scientific conferences, medical education and marketing activities) because these are governed by regional and national legislation or codes (e.g. EFPIA code of practice [8], FDA regulations [9]). As with the GPP guidelines, GPCAP focuses on the presentation of all types of company-sponsored research and the specific challenges surrounding this, rather than investigator-sponsored or investigator-initiated trials or research (where companies have no role in their presentation or publication), although many of the principles also apply to the presentation of other types of research at scientific meetings. The aim of GPCAP is therefore to provide guidance on good submission and presentation practice for scientific and medical congresses, specifically addressing certain aspects where current publication guidelines are inadequate.

## Methods

These recommendations were developed after informal discussions among a group of individuals who have wide experience of working with authors to develop abstracts, posters and slides for oral presentations reporting company-sponsored research. The main impetus for this article arose from a meeting regarding GPP3 updates (with which some of the authors had been involved). Prior to this meeting, two authors had noted that even the revised GPP3 guidelines contained limited advice for conference abstracts and presentations. Meeting participants discussed the requirement for clearer guidance and formed a working group to address this gap. At this point, invitations to join the group were extended to potential authors known to have previously presented relevant research at meetings of the International Society of Medical Publication Professionals (ISMPP) or had a known interest in conference presentations. This also ensured a broader global representation and improved the balance between pharmaceutical and medical communication agency representation. The authors all work or have worked for pharmaceutical companies and/or medical communication agencies (see the ‘Competing interest’ section for specific details). After a search for recommendations and guidelines on this topic revealed nothing specific (either in ICMJE or in a search on EQUATOR), the authors developed an initial outline for this article; individuals worked on pre-agreed sections and then a collective review of the full draft, comprising all sections was completed (see ‘Authors’ contributions’ for specific details). The resulting article was posted as a preprint on PeerJ [10] on 19 October 2017 for open comment. All comments received (and their responses) can be seen with the preprint on the PeerJ website. These comments were used to revise the recommendations. Some authors invited informal consultation from colleagues, and a courtesy legal review, as appropriate, was completed to ensure compliance with employee company policies. The copyright section was reviewed specifically for appropriate interpretation of copyright law. In addition to the preprint, 65 medical societies and medical conference sites, and two for-profit companies that run conferences on behalf of societies, were contacted for comment via contact emails listed on their websites or via ‘contact us’ options found on their websites. The societies and conferences and conference service companies were selected by recommendation from within the author group, to ensure balance across therapeutic areas, geography and variety of website submission sophistication. Only one of these societies/companies responded. All comments received on the preprint by 10 July 2018 were collated and discussed, and this final version was generated. The preprint was viewed by 2769 unique visitors and downloaded 3300 times between 19 October 2017 and 25 March 2019.

The recommendations are given here by topic, and so there is some overlap by intention, to ensure that all the key elements for any given topic appear together and allow readers to browse by topic.

## Recommendations

The following principles aim to cover the key areas relevant for submissions to any research-based conference.

- Author listings should reflect those who did the research and can take accountability for its conduct, and for the analysis and interpretation of the findings. Criteria for authorship of conference abstracts and presentations should generally be the same as those for full publications, although there can be occasions where local presenters may be included as authors, for example, where a conference requires a presenter to be listed as an author.
- All authors should be involved in the development, and approve the final version, of any abstract, poster or slides that bears their names. For studies involving large numbers of researchers it may be most efficient for a subgroup of those involved in the studies to develop conference abstracts and presentations (similar to the use of a writing group to develop publications from large studies).
- Posters and slides should list key contributors and describe their contributions to the research and development of the presentation.
- Study registration numbers (e.g. ClinicalTrials.gov, EudraCT, PROSPERO) should be included on abstracts, posters and slides.
- All sources of funding for the research and its presentation, and any author conflicts of interest, should be disclosed on posters and slides, on the conference submission site, and if space permits, on abstracts.
- Any medical writing support and associated funding should be acknowledged on posters and slides, on the conference submission site, and if space permits, on abstracts.

**Fig. 1:**
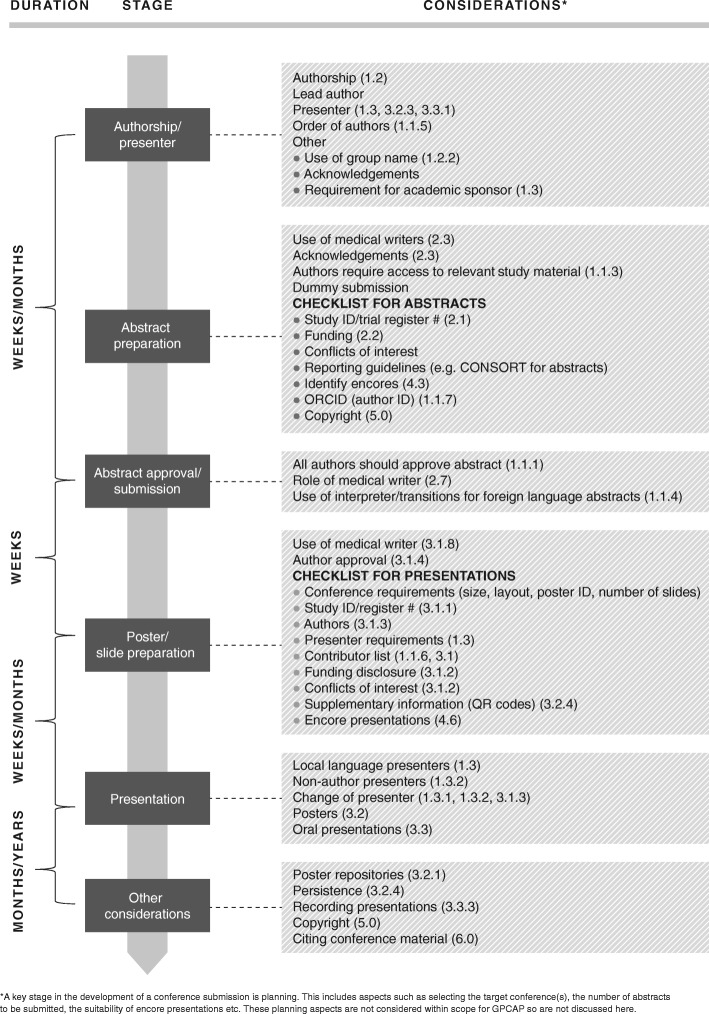
Roadmap of recommendations following abstract and presentation development stages

These recommendations are mapped against the development of an abstract and subsequent conference presentation workflow in Fig. 1, referenced by section number.

### Recommendations for conference organizers

Conference organizers should:

- encourage the inclusion of contributor lists on posters and slides;
- include a field for trial registration details on abstract forms (outside the word or character limit) and publish this information with the abstract;
- include a field for sponsor information on abstract forms (outside the word or character limit) and publish this information with the abstract;
- include a field for disclosing medical writing support on abstract forms (outside the word or character limit) and publish this information with the abstract;
- use ORCID identifiers (individual researcher identifiers [11]) to identify authors and presenters;
- not set arbitrary limits on the number of authors, and permit the use of study group names; and
- distinguish between authors (meeting the ICMJE criteria) and any additional individuals (who are not authors or contributors) included in the submission, for example, as a result of a requirement for a society member to sponsor submissions. With limited space in any printed book of abstracts, this information might be restricted to appearing with the online version of the abstract.

### 1.0 Authorship

#### 1.1 Authors

1.1.1 The author listing on conference abstracts and presentations should reflect the people who did the research or contributed substantially to the design of the study or to the interpretation of the results, and who were involved in the development of the presentation and who are willing to take responsibility for the findings. Authorship and author order should be agreed by all authors (see 1.1.5 for factors to consider). While the authorship criteria recommended by the ICMJE are widely used for journal articles [2], GPP3 recognizes that it may be necessary to adopt slightly different criteria for conference abstracts and presentations [5]. For example, while all named authors should review (at least once), approve the content of abstracts and presentations and be willing to take responsibility for the findings, it may be impractical to expect all authors to contribute to drafting and critically revising abstracts in the same way as for full manuscripts, because of the abstract brevity, time constraints, etc. There is an argument for limiting the authors to a number that can meaningfully comment and review an abstract (see 1.2.1) and using a study group to identify others involved in the wider study. Our collective past experience indicates that it becomes impractical for everyone to be involved in a group with more than 10 authors, which is also the maximum number suggested by GPP3 [5].

1.1.2 Authorship criteria for all anticipated journal articles and *primary* conference presentations should, ideally, be agreed at the start of the research, and author listings for subsequent secondary abstracts and presentations should be finalized well before work starts on the secondary material [12]. As with journal publications, whatever criteria are used to determine authorship should be applied equally to all authors, regardless of whether they are company employees, contractors, independent clinicians, researchers or consultants.

1.1.3 Authors and contributors should have access to all relevant study materials and data to permit them to understand the research findings. Abstracts may need to be developed soon after results are analysed and before a final clinical study report is available. In such cases, authors should always have access to the protocol, statistical tables and any other information necessary to discuss and develop the planned abstract and presentation.

1.1.4 If individuals are authors on abstracts and presentations written in languages in which they are not proficient, companies should work with them and offer whatever reasonable assistance is required to permit them to discuss and review material effectively (e.g. to provide translations for the authors, or a discussion with an interpreter or local investigator/presenter who can read and explain the text). Authors may also choose not to be listed for such a conference abstract and presentation (see also 1.1.6).

1.1.5 Whatever convention is (or will be) used to determine the order of authors on the related full publications in journals should generally also be used to determine the order of listing on conference abstracts and presentations. The final order should be agreed by all authors; however, conference requirements (e.g. listing the presenting author first) must be respected. In cases where first or last co-authorship is requested, the conference organizers should be contacted for guidance.

1.1.6 While the authorship of conference abstracts and presentations should accurately reflect those who were involved in the research, individuals who meet the ICMJE authorship criteria (and may be listed on a subsequent full publication) may choose not to be listed for a conference abstract and presentation (e.g. if they are unable to review and/or approve the material within the deadline). While this individual choice should be respected, significant contributions to the research should be acknowledged where possible; that is, in a contributor list included on the presentation.

1.1.7 Conference organizers should encourage the use of ORCID identifiers to identify authors on abstracts and presentations, to avoid ambiguity between authors with similar or identical names. (Note: many journals and institutions now require authors to include their ORCID identifier at manuscript submission.)

#### 1.2 Contributors/study groups

1.2.1 We encourage conferences (and company sponsors) not to limit the number of authors (or contributors) who may be listed on an abstract or presentation, because this practice may prevent the author list from accurately reflecting who did the work. However, named authors should be limited to those who have actively participated in the development of the abstract (see 1.1.1). GPP3 recommends an author group of fewer than 10 [5]; above this number, naming a study group may be a more practical approach. Likewise, if the source data come from a study, and the authors involved in that study meet authorship criteria, then the use of a study group name is strongly recommended.

1.2.2 Study group names may be helpful to acknowledge contributions to projects involving a large number of people, in addition to named authors who have contributed both to the research and to developing the presentation. Inclusion of a study name, either in the title or by including a study group in the author listing, will facilitate linkage of conference abstracts and presentations with journal publications. However, this should not be a substitute for including a unique study identifier such as a registration number for clinical trials (e.g. ClinTrials.gov or EudraCT numbers), which is a more reliable linkage method because these can be used as search terms in relevant databases. Provision should be made for study group membership details to be added during abstract submission and made available via the conference website once an abstract has been accepted.

#### 1.3 Presenters and society sponsors

1.3.1 While the ICMJE criteria are a useful starting point for determining authorship, they were not designed for conference abstracts and presentations. Therefore, in certain circumstances, and if all authors agree, it is permissible for somebody who does not (or will not) meet the ICMJE authorship criteria for a journal article to present findings at a conference. For example, a local presenter may be included (preferably in a contributor list and not as an author) if the authors of the conference presentation will not attend a particular meeting, do not speak the language required or are not members of the academic society hosting the meeting. This local presenter, for example, could be an investigator who recruited patients but did not contribute to the study design or interpretation of data and will not be involved in developing journal articles. In the contributor list, this person should be designated as ‘presenter’ to clarify their role. However, if the conference requires that only authors can present, then the new presenter will need to be added to the author list.

1.3.2 Abstract authors (including company authors) attending a conference should always be preferred as presenters over non-author presenters. In cases where an author is not available to present, and the conference acquiesces to a non-author covering the presentation, the non-author presenter should be familiar with the research design and findings and have a good knowledge of the subject area in order to respond to questions about the presentation even if, unlike the authors, they cannot take direct responsibility for the research. An appropriately qualified individual from the sponsoring company (e.g. Medical Director) could present study findings if authors are not available; however, individuals with a commercial role in the sponsoring company (i.e. sales or marketing) should not act as non-author presenters.

1.3.3 All those listed as authors on an abstract or presentation must be able to take accountability for the research (following the spirit of the ICMJE recommendations). Therefore, if conferences require a society member to sponsor a submission, and none of the authors or study investigators is a member, this sponsorship role should be distinguished from that of the study authors if the sponsor/member was not involved with the research. If an existing author happened to be a society member, then no such distinction would be necessary. If the conference wishes to list the society sponsor, then this role should be indicated on the abstract (e.g. by an asterisk) and in a contributor list (not the author list) on the presentation.

**Fig. 2:**
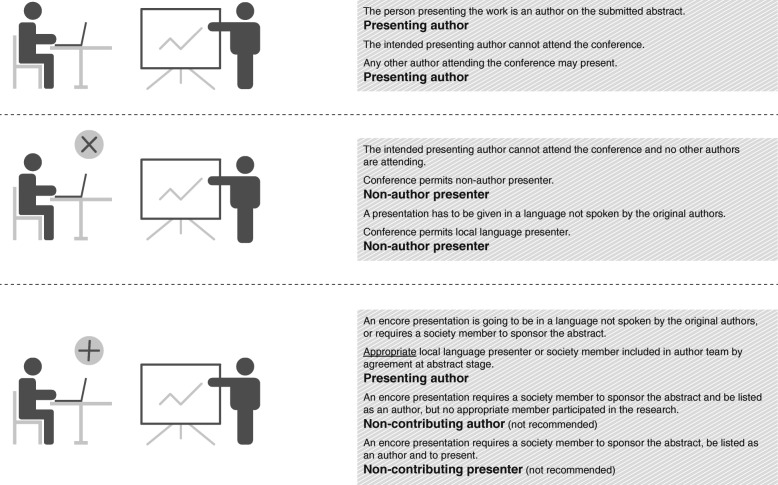
When is a presenter not an author? Different roles possible for authors and presenters of conference presentations

Figure 2 illustrates some scenarios to differentiate between authors and non-author presenters.

### 2.0 Conference abstracts

2.1 To facilitate linkage between conference abstracts and presentations, and subsequent publications, abstracts should include a study identifier such as a registration number (for clinical trials), study name, protocol number or grant number. To encourage this, conference organizers should require this information in a specific field on the submission form and publish it with the abstract.

2.2 Abstracts describing company-sponsored research should always name the sponsor and all funding sources (if more than the sponsor).

2.3 Authors or sponsoring companies may involve professional medical writers to support authors in the drafting of abstracts. All authors should agree to these arrangements and work closely with any writers and approve the final version. Space limitations on abstract submission sites usually preclude writing support acknowledgement. Conference organizers should consider requesting this information and publishing it with the abstract.

2.4 We encourage conference organizers to consider the requirements of reporting guidelines when setting limits on the length of abstracts. For example, CONSORT for Abstracts suggests that around 300 words may be needed to adequately report randomized clinical trials [7].

2.5 We also encourage conference organizers to maximize the available space for content in abstracts by not counting authors, affiliations, trial registration numbers and sponsor acknowledgments towards the word or character limit.

2.6 Most conferences will not consider reports of findings that have already been published in full (i.e. in a peer-reviewed journal). This requirement must be respected and, even if permitted, presenting findings after their full publication should be avoided. However, abstracts presenting findings or novel analyses that are not included in a full publication may be submitted if the conference permits this. In situations where a journal article is in preparation at the same time as abstract submission, subsequent submission of the article may overtake the abstract in acceptance, at which point the conference needs to be advised, and the journal also, to avoid issues of prior data release. It may be necessary to withdraw the abstract, or it might be possible for the journal and conference to come to a mutually acceptable arrangement regarding either delay of the article or amendment to the intended presentation. Posting summary results on a trial register (e.g. ClinicalTrials.gov, EudraCT) or a clinical study report to meet regulatory requirements is not regarded as full publication by the ICMJE [2] and should not prevent subsequent presentation at conferences.

2.7 As conference submission requirements become more detailed (and therefore labour-intensive), conference organizers should acknowledge that it is acceptable for the abstract submission process to be completed by a third party (e.g. a medical communications company) on behalf of the submitting author, with that author’s permission. Where feasible, the submission might be checked by the submitting author prior to the actual submission; however, there are some sites where submission has to be completed in one sitting, and on other occasions, time differences (and time pressures) may make this impractical.

### 3.0 Conference presentations (posters and slides)

#### 3.1 General considerations

3.1.1 Study identifiers (e.g., trial registration numbers) should be included on presentations to improve linkage between conference presentations and subsequent publications (see also Section 4).

3.1.2 All funding sources for the research, any assistance with the presentation (e.g. medical writing support, editorial assistance or design) or support for conference attendance and authors’ conflicts of interest should be clearly disclosed on posters and slides. For posters and slides, such disclosures should be clearly legible (i.e. not significantly smaller or lighter-coloured than the main text).

3.1.3 Author listing and order on posters and slides should be the same as that on the abstract. Authors should not be added to a presentation after the abstract is accepted. However, if an author is unavailable to work on a presentation after abstract acceptance, their name may be removed from the author list but their contribution (to the study and/or publication) should be acknowledged. If an author other than the first-named author is to present, this should be indicated without changing the author order. The principle is to retain the same information about authors as on the abstract for ease of identifying the related presentation. Similarly, the title of the presentation should not be changed after submission; thus, the titles of the abstract and poster or slides should be identical. [If someone not on the author list is to present, and this is known in time for poster preparation, the relevant name could be added as a footnote, or close to the author list thus: (Presenter: J. Doe, ABC Institute, City, Country).]

3.1.4 All named authors should contribute to the development of, and approve, the presentation (see 1.1.1). Authors should be given sufficient time for presentation development and review. Making significant changes to posters or slides after all-author approval should be avoided. If changes must be made after approval, the actual final version must be sent to all authors. As with journal articles, for large studies, it may be most efficient for a subgroup to coordinate the development of a presentation (similar to a writing group for an article). This should be considered when deciding authorship.

3.1.5 Each author’s contributions to the study and to the development of the presentation should be listed.

3.1.6 Conference presentations should include a list of contributors who have made a significant contribution to the research or the presentation, regardless of whether they are listed as authors or attending the meeting. Ideally, permission for such acknowledgment should be sought in writing.

3.1.7 Because abstracts are usually submitted several months before a conference, they may contain interim or preliminary findings. Therefore, by the time of the conference presentation, some details may have changed. If research findings change substantially between abstract submission and conference presentation and affect the conclusions of the research, we recommend that authors alert the conference to this discrepancy. This is particularly pertinent in the case of oral presentations (because abstracts are typically selected for oral presentations based on the impact of the findings). Regardless of whether the new data change the conclusions of the research, we recommend indicating (e.g. in a footnote) any data that are different from those on the accepted abstract.

3.1.8 Authors or sponsoring companies may involve professional medical writers in the production of posters and slides. Authors should agree to these arrangements and work closely with any writers, editors and/or designers throughout the development of the presentation. Such support should be disclosed on the presentation, along with source(s) of funding (see also 3.1.2).

#### 3.2 Posters

3.2.1 While there are platforms where posters can be made permanently available (e.g. on conference websites or platforms such as F1000 Research), some journals regard this as prior publication which may jeopardize full publication. Authors should therefore check the policies of their target journal(s) and of the sponsor or funder before agreeing to a poster being publicly posted.

3.2.2 Posters are not peer-reviewed by conferences and may not describe all aspects of the research. Posters should therefore not be viewed as a substitute for a full article in a peer-reviewed journal. However, if a poster is publicly available (and, ideally, searchable via an indexing system or DOI), it may be cited until the full publication is available, although some journals consider citation of posters as unpublished information rather than full citations. See Section 6 for citation best practice.

3.2.3 The lead author should be given the first option to attend the poster session(s), but this role may be taken by other authors or a local presenter (if no author can attend or if no authors can present in the language of the conference). The poster presenter should ideally be agreed before the abstract is submitted, although it is understood that circumstances may change by the time of the actual conference (see 1.3.1).

3.2.4 While disclosures, funding sources, acknowledgements and contributions should be clearly noted on the main poster, supplementary sources can be used to expand on these if there is not enough room for detailed information, and may be accessed via a QR code (or similar link). Such content should normally be available until the research is published, in full, in a journal (at which point the link should be deactivated). If QR codes (or similar technology) are used to provide copies of the poster or to link to other scientific content, these should only be available to conference attendees, unless the conference elects to make the posters freely available after the conference. Links for the QR codes may be time-limited to close once the conference is finished. Supplementary materials may include translations. Supplementary material should be provided under the same usage conditions as the poster and indicate who is the copyright holder or licensee.

#### 3.3 Slides for oral presentations

3.3.1 While the lead author is normally expected to present study findings at conferences (and is given the first option to do so), this may not be possible due to local language requirements, availability to travel, or personal circumstances, etc. If the lead author chooses not to present study findings, another author may give the oral presentation. If none of the named authors is available or able to give the presentation, a non-author presenter may present the findings if all authors agree to this and the conference permits it (see also 1.3.1 and 1.3.2). The presenter should be agreed before the abstract is submitted (and only changed if that person becomes unavailable). The lead author should discuss the contents of the presentation and the interpretation of the findings with the presenter (and co-authors, if possible) before the conference to ensure the authors’ views are correctly represented.

3.3.2 If a non-author presenter gives a presentation on behalf of the named authors (or study group), this should be indicated at the beginning of the presentation. The presenter’s conflicts of interest should be noted on the disclosure slide.

3.3.3 Recordings of oral presentations may be posted online by conference organizers but, as with posters, care should be taken to ensure this does not jeopardize full publication in a peer-reviewed journal. Slides alone (without the accompanying talk or speaker notes) may be hard to interpret and not provide full context, so care should be taken if these are made publicly available. As with posters (see 3.2.4), online sources may also be considered to host supplementary materials for presentations if they are made available after the presentation. If slides are made publicly available, this should not occur until after the presentation has been given and should only occur with the agreement of all authors and sponsors, who will need to consider any restrictions around the posting of the data and possible ‘prior publication’ concerns for later use (see 6.1.2).

3.3.4 Some scientific meetings offer Continuing Medical Education (CME) credit for attendance at oral presentations. Local regulations and requirements of the accreditation body for this must be respected.

### 4.0 Encore abstracts and presentations

4.1. It is permissible to present the same research findings at more than one conference if both the first and subsequent conferences allow this. This practice may be referred to as an ‘encore’ (or more specifically an encore abstract or encore presentation). However, presentations of the same findings to the same audience should be avoided.

4.2 Although encore abstracts are not considered to be redundant publications (unlike publication of the same findings in more than one journal), some conferences elect only to accept findings that have not been presented at other conferences, and such requirements must be respected.

4.3 When considering encore abstracts, the authors and sponsoring company should decide whether it is most appropriate to submit identical abstracts to multiple conferences or whether it is better to emphasize different aspects of a trial (e.g. those of interest to different audiences). Use of study identifiers can help identify that multiple conference abstracts and presentations are from a single study. However, to avoid any confusion, we recommend that encores should be specifically identified as such (e.g. by stating that the presentation is an ‘encore’ and listing where previous abstracts of all or some of the findings were presented) (see also 4.4 and 4.6). We also recommend that previous presentations should be listed on the presentation, if accepted.

4.4 Conference organizers should consider including a means of identifying encore abstracts (e.g. including details of prior presentations) on the abstract submission form. This information should not be included in the abstract word or character count.

4.5 Addition of new data to a previously accepted abstract may not necessarily constitute a new abstract: conference guidelines should be consulted to confirm if this is acceptable. If no specific guidelines are provided, then as a general guide, if the new iteration adds any new data other than an update on analyses already contained in a previous abstract, then the new iteration should be regarded as a new abstract.

4.6 Where encore abstracts, or updated abstracts that include previously presented data, are accepted, their presentations should indicate that this is not the first time of presentation, for example, by a statement on the poster or slides such as “Data/some data first presented at [conference name and date]”.

4.7 Encore checklist: When deciding whether to submit an encore abstract to a conference to reach different audiences, authors and study sponsors should consider the following points.

- What is the overlap, if any, with the audience of the earlier conference (e.g. in terms of region, specialism or profession)?
- Are there any differences in the licensing status of any products mentioned in the presentation between the first and subsequent conference locations? For example, if the first presentation occurred in a region where a product is licensed, but later presentation(s) will take place in a region where it is not yet licensed, this fact may need to be reflected. For international meetings, remember that participants will attend from several regions, so the licensing status in different countries should be clarified.
- Presentation at multiple meetings might delay and/or potentially jeopardize the full publication of research in a peer-reviewed journal. Companies should consider whether resources would therefore be better spent on ensuring a timely submission to a journal rather than preparing several encore abstracts and presentations.

### 5.0 Copyright considerations

5.1 Copyright transfer or publishing licence agreements that are executed during the abstract submission process are common when abstracts are to be formally published (e.g. in a conference-specific journal issue). These agreements relate only to the abstract, not to any subsequent presentation, unless explicitly agreed otherwise.

5.2 Copyright in a presentation is normally held by the authors, unless they have assigned it either to the conference or the sponsoring company. Re-use of a poster (at a subsequent meeting or in another format, such as a poster book or handout) normally requires permission from the copyright holder(s). It may therefore be simplest for authors to assign usage rights to the sponsor company if encore presentations or other types of re-use are planned. If a company author is included, then the copyright for that individual’s contribution rests with the company (not the employee).

5.3 If a conference wishes to acquire usage rights for abstracts, slides, or posters, we recommend that the conference offers an open access option under a Creative Commons (CC) licence. We encourage the use of the least restrictive CC-BY licence, which will allow authors and sponsoring companies the usage rights for subsequent presentations, as well as future publications. If presentations contain third-party material to which the authors do not hold copyright, it should be the responsibility of the conference organizers to clear rights for any further usage. The authors cannot be expected to anticipate the future use of materials by the conference organizers.

5.4 As for any publication, permission must be sought for use of third-party copyrighted material (e.g. a figure) in a presentation (and again for any encore presentations). Material should not be altered simply to avoid having to obtain permission from the copyright holder.

5.5 Peer-to-peer presentation at a scholarly conference by a researcher is generally considered to be fair dealing (UK) [13] or fair use (USA) [14], which does not require copyright permission. Any other use of a presentation by a company outside the conference will most likely be considered commercial use, for which permission from the rights holder(s) will be necessary.

### 6.0 Citing conference material

6.1 References (or citations) in scientific texts provide readers with source or background material and are used to justify or support statements. To be useable, the referenced material must be both permanently accessible and reliable; therefore, citations to full publications in journals that apply rigorous peer review are the ideal. However, if citations are needed for research that has not yet been fully published in a peer-reviewed journal, abstracts that have undergone scientific review (and on the basis of that have been accepted for presentation by a conference) may be cited, especially if they have also been published in a journal and are therefore permanently accessible and discoverable. Abstracts should not be cited after the full (primary) publication has been accepted by a journal.

6.2 Posters and slides are not peer-reviewed by conferences and are often not permanently or widely accessible or discoverable. Citations to posters or slides should therefore be avoided (see 6.1). However, if a poster or slide set is publicly available (and, ideally, discoverable via an indexing system or DOI), it may be cited until the full publication is available (although some journals consider citation of posters or slides as unpublished information rather than full citations). Authors and sponsor companies should ensure that publishing posters or slides online does not jeopardize full publication in a peer-reviewed journal.

6.3 To avoid citing conference posters or slides, companies should consider other dissemination routes such as listing findings as ‘Data on File’ (i.e. an unpublished data package held by the pharmaceutical company, which then should be supplied to anyone requesting those data).

6.4 If specific findings that were presented at a conference are omitted from a journal article (e.g. because of space constraints), they could be made accessible as supplementary material.

## Discussion

These recommendations summarize the authors’ collective experience with a view to outlining the underlying principles for best practice and providing guidance on the practicalities for navigating conference requirements. We did consider whether some of our recommendations could be accomplished by amendments to company–author agreements, but decided that such recommendations for ‘good practice for author agreements’ were beyond the remit and scope of this article and that GPP3 [5] adequately covers this aspect of author–sponsor relationship. Many of these recommendations are drawn from the working group’s experience across a variety of disease areas and conferences. However, this is also a limitation, in that by the nature of the authors’ work, their experience lies in conferences and conference submission systems with strong industry involvement. We believe that these recommendations could be applied to any type of scientific/medical conference and are as relevant to academic research as to company-sponsored research. Conferences maintain their value to the scientific community by covering the latest research and providing a forum for discussion: this value must not be lost due to lack of transparency or ethics in the preparation and presentation of the new data. By following these recommendations, industry professionals, authors and conference organizers will improve consistency, transparency and integrity of publications submitted to conferences worldwide.

It is earnestly hoped that future input from conference organizers and societies, as well anyone involved in submitting research to conferences, will augment and strengthen these recommendations. We therefore welcome feedback via the website (https://gpcap.org).

## Authors’ information

Cate Foster, Jackie Marchington, Steve Banner and Nina C Kennard work for medical communication agencies that provide professional medical writing or editing services to not-for-profit and for-profit clients.

Elizabeth Wager is self-employed and provides training, consultancy and editing services on medical publishing and publication ethics for pharmaceutical companies, medical communication agencies, publishers, universities and academic societies.

Mina Patel and Antonia Panayi work in Global Medical Affairs functions within the pharmaceutical industry.

Rianne Stacey worked for a medical communication agency (see above) during the majority of the time the work was done and now works at Jazz Pharmaceuticals, Oxford, UK, in a Global Medical Affairs function within the pharmaceutical industry.
